# Is Tanzania’s economic growth leaving the poor behind? A nonlinear autoregressive distributed lag assessment

**DOI:** 10.1371/journal.pone.0270036

**Published:** 2022-07-08

**Authors:** Valensi Corbinian Kyara, Mohammad Mafizur Rahman, Rasheda Khanam

**Affiliations:** School of Business, University of Southern Queensland, Toowoomba, QLD, Australia; Kedge Business School, FRANCE

## Abstract

Most developing economies have recently experienced significant economic growth without corresponding substantial poverty reduction and improved population wellbeing. This paper uses a nonlinear autoregressive distributed lag model to explore the growth-poverty relationship in Tanzania using annual time series data on per capita consumption expenditure, real GDP, GINI index, and unemployment from 1991–2020. To explore the causality among the variables and long-run asymmetry between per capita consumption expenditure and economic growth, the study employs Granger causality and Wild test respectively. The results confirm the presence of long and short-run asymmetric behavior of economic growth. Besides, in the short-run, the Granger causality test supported the feedback hypothesis between economic growth and consumption expenditure, and the unidirectional hypothesis from income inequality and unemployment to consumption expenditure. In the long-run, unidirectional causality was observed from consumption expenditure to both economic growth and unemployment. The study submits that while economic growth exhibits poverty reduction features, growth alone is not sufficient to alleviate poverty because the interaction of income inequality with economic growth dampens the poverty-reducing effects of economic growth. Therefore, economic growth has a significant explanation for poverty but not all about the evolution of poverty. The study opens policy perspectives with wide international relevancy as outlined in the policy implication section.

## 1.0 Introduction

Increasing economic growth has received extensive discourse in research and government following its perceived inherent potential to alleviate poverty and improve populations’ wellbeing. An increase in economic growth is normally considered good news, especially for the country’s poor, while vice versa is true. Nevertheless, in recent years, it has been observed that economic growth, especially in developing countries does not elicit a corresponding poverty reduction [1–3]. Such a mismatch between growth and poverty is generally referred to as a growth-poverty dilemma.

This research queries the dilemma of growth-poverty mismatch in developing nations by investigating and presenting empirical evidence as a contribution to narrowing the gap in growth-poverty literature. The study is unique because it brings a new perspective that extends and enriches previous growth-poverty empirical studies by investigating whether economic growth is sufficient for alleviating consumption deprivation poverty and delivering improved quality of population’s wellbeing in developing economies. Most of the previous studies [4–6] dwelt on the question of whether economic growth ameliorates the incidence of poverty. Furthermore, most of the previous studies used cross-sectional data and linear autoregressive methods to assess the growth-poverty nexus; very few studies have employed time-series data. Therefore, the current study employs time series data and a nonlinear autoregressive distributed lag approach to add a new methodological perspective to the literature. The findings of the study affirm that while economic growth carries feasible kernels for poverty alleviation, growth alone is not sufficient for poverty alleviation and improved population wellbeing. Factors such as income inequality and unemployment tend to dampen the poverty-alleviating impact of economic growth, thereby aggravating the population’s quality of life.

While the scenario of growth-poverty mismatch is not limited to developing economies, its manifestations are more pronounced in developing countries. It is from this background, therefore, that the authors are motivated to investigate empirical evidence on the growth-poverty dilemma, using Tanzania as a case in point and focusing on consumption deprivation poverty; an area that is still under-researched.

Economies of most developing nations, such as Tanzania, contain inherent characteristics such as lack of access to meaningful employment, social and income inequalities, low capital formation, the rapid increase of population, high levels of inflation, the vicious circle of poverty, struggle over the rights and market of resources, and severe vulnerability to climate change. Such characteristics pose a serious stumbling block on the path toward the realization of the United Nations’ Sustainable Development Goals 2030. For instance, poverty alleviation is one of the key development challenges facing Tanzania since its political independence in 1961. The history of the country’s development strategies gives evidence of many, and rich policies formulated to spearhead growth for poverty reduction. Consequently, the Tanzanian economy has enjoyed an upward growth trajectory, especially over the last three decades, after the 1990s economic reforms which came at a near economic collapse in the 1980s. For instance, GDP improved from US$ 5.25 billion in 1995 to US$ 18.39 billion in 2005 and then up to US$ 61.14 billion in 2019 [7]. During the period 2009–2019, the economy has been growing at an average rate of 6.2% annually; where the highest growth was 7.67% in 2011and the lowest rate of 4.5% was registered in 2012 [7].

Despite the high and consistent economic growth in Tanzania, the fruits of increased economic growth have not reached rural and peri-urban areas where the majority of the real poor are hosted [2, 8–10]. For instance, the rapid growth has not succeeded in generating decent and adequate jobs; the average annual unemployment rate during 2010–2020 is 2.45% [7]. Rural poverty in Tanzania is much more pronounced, as compared with its urban counterpart. Most of the poor are in the rural areas where poverty-combating facilities such as access to quality medical care, basic education, reliable transportation, and clean water are noticeably missing in most rural settings. The ill-developed infrastructures especially in rural areas further complicate the poverty scenario in Tanzania and are not able to support the needed economic transformation. As a result, economic growth in Tanzania has marginally combated poverty among rural and agrarian households. Thus, the Tanzanian economy presents a unique situation because it has achieved high strides in terms of growth over the last three decades, but such rapid growth has not elicited a commensurate level of poverty reduction and improved wellbeing [1]. This scenario, therefore, calls for a systematic investigation into the nature and relationship between growth and wellbeing in Tanzania. Hence, the current study queries the uniqueness of the prevailing poverty-growth dilemma in Tanzania from the perspective of the population’s wellbeing.

It is widely considered that increasing economic growth comes with poverty alleviating effects [4, 5, 11]. Thus, a high real GDP is theoretically associated with a reduced incidence of poverty and an improvement in quality of life. Nevertheless, conditions such as unemployment and income inequality dampen the poverty-reducing impact of economic growth [4, 11]. As a result, the rising mean income is not benefiting everybody [12]. Moreover, poverty is most manifested in developing economies, and it is affirmed that managing income inequality is one of the important approaches to combating poverty.

This study has two primary objectives. First, to investigate empirical evidence and the importance of economic growth for alleviation of consumption deprivation poverty and improved population wellbeing in Tanzania. Second, to investigate the nature and impact of the interaction of economic growth with income inequality on the quality of the population’s wellbeing. While such investigations could take a comparative approach, e.g., a cluster of East African or sub-Saharan countries, scrutinizing the impact of growth on consumption deprivation in Tanzania is germane due to the country’s unique political and economic history. Unlike other sub-Saharan African economies, the Tanzanian economy is shaped by unique macroeconomic and political reforms that shape the current alignment, nature, and magnitude of doing business in Tanzania. These include the Ujamaa development policy—communal self-reliance policy, Kiswahili language which unified all the ethnic groups in the country, the existence of reasonable democratic rule of law and smooth transition of power, etc.

To probe the discourse, a time series data on the rate of growth of real economic growth, GINI index, and unemployment for the period 1991–2020 are analyzed using a nonlinear autoregressive distributed lag approach and Wald test to explore symmetry among the variables. The choice of the period i.e., 1991–2020 is based on first, the economic progress registered by the Tanzanian economy following the 1990s economic reforms. Second, the period is determined by the availability of reliable data for the chosen variables.

The current study makes a distinctive contribution to the growth-poverty literature because it is one of only a handful of studies that explore empirically the relationship between growth and consumption expenditure in the presence of income inequality and unemployment in developing countries. Besides, to the best understanding of the authors, the study pioneers such investigation for the first time using Tanzanian data and methodologically it is the first of its kind in sub-Saharan Africa where consumption deprivation and poverty are rife. While the empirical assessment is based on Tanzanian data, the findings are of great significance for developing economies because the knowledge of the growth-poverty nexus and the impact of economic growth interaction with income inequality and unemployment can assist planners in conducting policy instruments to enhance household consumption expenditure and improve overall population’s wellbeing.

To attain the objective of the study, the remaining part of this study is organized as follows. After a brief literature review in section two, section three presents the study methodology, followed by a presentation of empirical estimation findings and discussions in section four. Section five outlines key policy implications based on the findings and concluding remarks.

## 2.0 Brief literature review

The 2030 Sustainable Development Goals 1 and 10 seek to alleviate all forms of poverty and make significant strides in reducing inequality [13]. Various approaches are being taken to realize poverty reduction and improved livelihood. The growth-poverty-inequality literature suggests some aspects that researchers and practitioners must pay attention to when assessing and addressing poverty and poverty-related issues. Considering the scope of this study and without trying to be exhaustive, we draw our attention to three of such aspects: inequality aggravates poverty, economic growth alleviates poverty, and growth-inequality-poverty exhibits inseparable triangular relationships.

First, most studies have observed that inequality aggravates poverty because it reduces the level of disposable income, thereby limiting individuals’ purchasing power, and ultimately leading to consumption deprivation. Proponents of this school attest that inequality and poverty are closely netted together in such a way that strategies to end income inequality will also lead to poverty reduction [11, 14–17]. For instance, some researchers [17] assessed the impact of financial development, income inequality, and economic growth on poverty in India using time series data for the period 1970–2015. The linear autoregressive distributed lag model bound testing procedure delivered evidence that while economic growth and financial development help to alleviate poverty, income inequality and inflation aggravated poverty significantly. Therefore, different countries may experience rapid economic progress over a prolonged period but the rate at which such growth translates into poverty reduction and improved livelihood is among other factors dependent on the parallel efforts in place to curb income inequality [4, 17, 18].

Second, there is a cluster of studies propagating higher levels of economic growth as a tool for poverty alleviation: Sustained economic progress approach, commonly rerefer to as the trickle-down economics approach, is frequently cited in the literature as among the traditional approaches to address poverty [5, 17, 19–22]. However, the results from these studies exhibit variation across regions, countries, and even disparity across various parts of the same country–e.g., rural vs. urban. This suggests that different regions have different underlying conditions in such a way that the same rate of economic growth produces a varied impact on poverty and people’s wellbeing. For instance, to this end, some scholars [8, 10, 23, 24] attest that in the recent years most of the sub-Sahara African countries have experienced wealth without prosperity; the rapid economic progress is not accompanied with household poverty reduction and improved quality of life.

Third, some studies attest that income inequality, growth, and poverty exhibit inseparable triangular relationship, i.e., subject to poverty income inequality and growth can be either positive or negative depending on the empirical approach employed, while subject to income inequality, poverty and economic growth are negatively correlated irrespective of the method employed [22, 25]. In this case, policies to address e.g., poverty must consider the inbuilt connection between poverty, inequality, and growth [4, 26, 27]. Therefore, these findings confirm that poverty is a multi-pronged approach issue, and its alleviation calls for a multi-dimensional strategy. A single approach to poverty alleviation will fail to yield the desired results.

The current study contributes to the debate on the growth-poverty-inequality debate by presenting an empirical assessment of economic growth, income inequality, and consumption deprivation in Tanzania. While empirical studies on consumption deprivation poverty are still limited in sub-Saharan Africa, to the best of the authors’ understanding, this is the first study of its kind focusing on Tanzania and using the NARDL approach for analysis.

## 3.0 Methodology

Taking into consideration the goal of this study, a linear model is not an ideal model because of the possibility that our data may comprise some inherent nonlinearities. It has been affirmed that nonlinear autoregressive models provide a better fit for volatility as compared to the traditional linear autoregressive models which tend to impose unrealistic restrictions, culminating in biased inferences [28–30].

Therefore, the nonlinear autoregressive distributed lag (NARDL) mode is the most suited for our analysis because, first it allows for testing the responses of the explained variable to changes in each of the explanatory variables, and so makes it possible to build asymmetry line [31]. Second, NARDL lends a hand in differentiating the long-run and short-run effects of changes in independent variables in the dependent variable. In this case, the model allows for ascertaining key features in the immediate reaction of the dependent variable following the shocks in the independent variable [31, 32]. Third, NARDL is prominent for its ability to handle both linear and nonlinear cointegration as well as accommodate multiple data series with different orders of integration [33].

### 3.1 Data and variables

This study employs annual time series data on per capita consumption expenditure (CE) as a proxy for consumption deprivation poverty, GDP growth rate (EG) as a proxy for economic growth, GIN index growth rate (IQ) as a proxy for income inequality, and total unemployment rate (UE)—a % of the labor force—to depict the proportion of total labor force willing and able to work but without work, all for the period 1991–2019.

**Fig 1:**
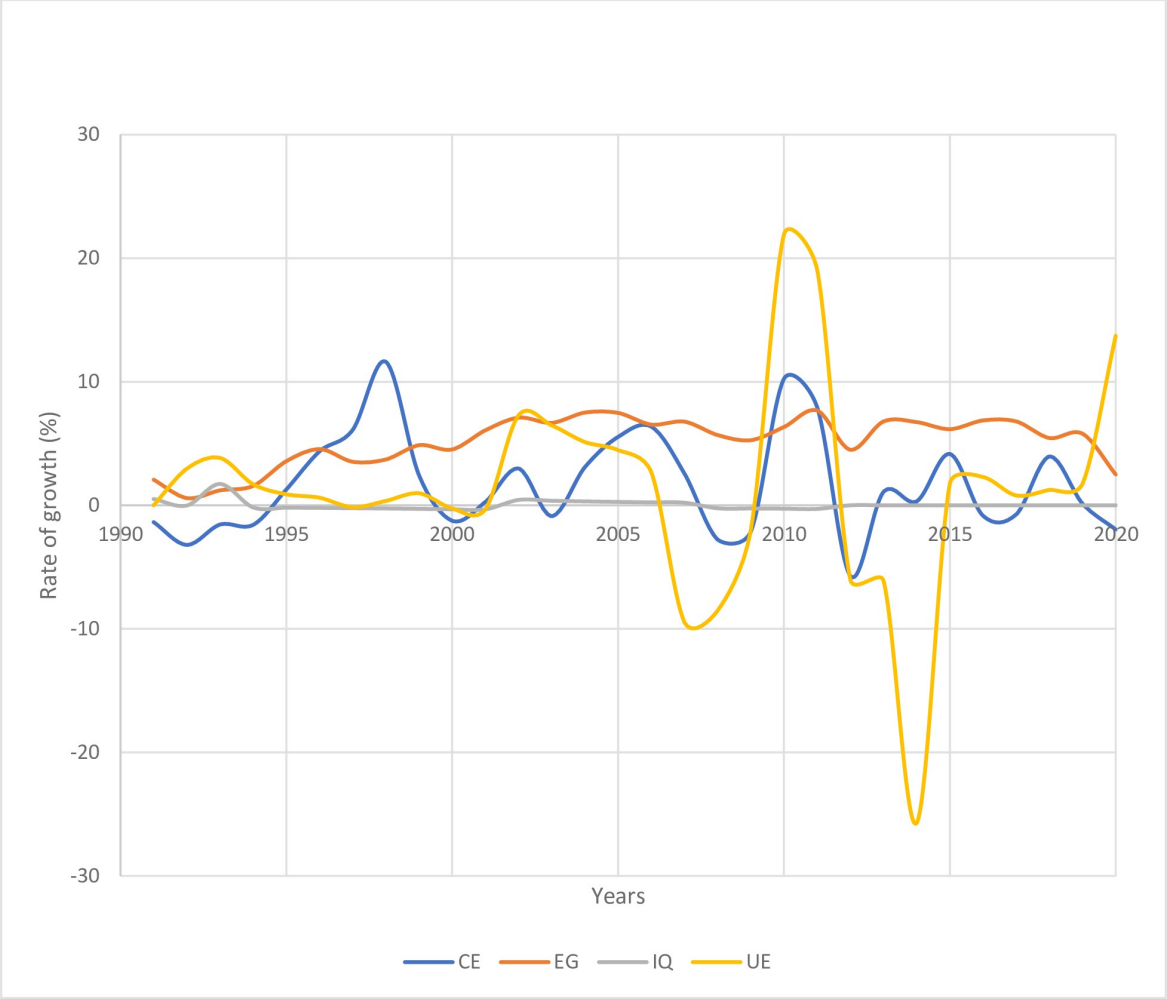
The rate of growth of GDP, unemployment, GINI index and consumption expenditure.

- **Per capita consumption expenditure.** The CE is employed here as a proxy for measuring consumption deprivation poverty. It is the best proxy for consumption deprivation poverty, and so measures of population’s wellbeing, because consumption expenditure among the poor is more reliably reported and more stable as compared to income [17, 34, 35]. Besides, CE as a measure of consumption deprivation poverty is analogous to the World Bank’s standard description of poverty as the inability to attain a minimum acceptable standard of living as quantified in terms of basic consumption needs [36]. Further, due to the inaccessibility to poverty headcount data for many countries, consumption expenditure has been widely used to measure consumption deprivation poverty as an alternative approach to cast insight on the overall scenario of poverty [4, 35, 37–39].
- **GDP growth rate.** GDP growth rate is widely used in the literature as a standard proxy for economic growth [40–43]. GDP is frequently considered an effective indicator of economic growth because it gives quantifiable information about the size and the performance of the economy. Thus, the real GDP growth rate gauges the health of the economy because an increase in real GDP is an indication that overall the economy is performing well. If the real GDP is falling, that is an indication of economic stagnation and or decline and the nation is not making economic progress. An increase in real GDP is expected to have a positive impact on per capita final consumption expenditure, and thus compact consumption deprivation.
- **GINI index.** GIN index, a well-celebrated measure of income inequality, is a statistical measure of income or wealth distribution among individuals relative to the entire country’s population [4, 44–46]. It is also referred to as the GINI index and it ranges from 0 (0%) to 1 (1%) such that 0 represent perfect equality and 1 perfect inequality. It is noted here that while the GINI index shows the income distribution among the population in a country, it, however, does not show its overall income [47]. Thus, a low-income and high-income country can exhibit the same GINI index for the index is only an indication of wealth distribution, not income level.
- **Unemployment.** Unemployment, measured as % of the labor force in a country willing and able to work but without work, indicates the extent to which the active population is inhibited from obtaining the reliable capability to access necessary income for improved livelihood [4, 48]. The description of each variable and data source is summarized in Table 1. *Fig 1: The rate of growth of GDP, unemployment, GINI index, and consumption expenditure*, is a graphical representation showing the trend of the variables. The horizontal straight line at the middle of the figure represents time in years. The blue trend line represents the consumption expenditure, which is the dependent variable. The other 3 trend lines depict independent variables, i.e., the dark-yellow line depict economic growth (EG) trend, the grey line depicts income inequality (IQ) trend, and the light-yellow line depicts unemployment (UE) trend. Overall, all independent variables exhibit upward trend, and they seem to be associated with the variation in the dependent variable.

**Table 1:** Description of variables and corresponding statistical data sources.

Variable	Description	Data source
Per capita consumption expenditure	Proxy for consumption deprivation poverty	[7] World Development Indicators
The growth rate of consumption expenditure (% annual)	The annual growth rate of per capita consumption expenditure	Computed by the Authors
GDP growth rate (% annual)	The annual growth rate of the economy	[7] World Development Indicators
GINI index	The measure of income inequality	[49] The global consumption and income project
GINI index growth rate (% annual)	The annual growth rate of income inequality	Computed by the Authors
Total unemployment	total labor force willing and able to work but without work*.	[7] World Development Indicators
Unemployment growth rate (% annual)	The annual growth rate of unemployment	Authors’ calculations

* Derived by multiplying the provided annual unemployment rate (%) by the WID provided total labor force data.

### 3.2 Empirical model specification

The study applies a nonlinear autoregressive distributed lag (NARDL) model to assess the relationship between consumption expenditure, GDP growth, income inequality, and unemployment in Tanzania during 1991–2020. In addition to the advantages of using the NARDL model outlined in section 3.0 above, we also observe that some previous studies have used the NARDL approach to assess asymmetry in financial markets [50], energy policy [51], uncertainty impacts [52], commodity pricing [53, 54], etc. Nevertheless, to our best knowledge, our study is the first one to apply the NARDL model for exploring the relationship between economic growth and consumption expenditure, to measure consumption deprivation poverty.

On the basis of previous empirical studies [32, 55, 56], the relationship among the variables is construed as follows:

$$CE_{t}=\beta_{0}+\beta_{1}EG_{t}+\beta_{2}IQ_{t}+\beta_{3}UE_{t}+e_{t}$$ (1)

Where CE is the per capita consumption expenditure growth rate, measuring consumption deprivation poverty; EG is GDP (economic) growth rate, IQ is the growth rate of income inequality, UE is an annual growth rate of unemployment, and β_i_ is a vector of long-run coefficients to be estimated.

In view of some previous scholarly works [12, 32, 56], to account for the asymmetries among the variables, Eq 1 can be expressed as:

$${\mathrm{CE}}_{t}=\theta_{0}+\theta_{1}{\mathrm{EG}}_{t}{}^{+}+\theta_{2}{\mathrm{EG}}_{t}{}^{‐}+\beta_{3}{\mathrm{IQ}}_{t}+\beta_{4}{\mathrm{UE}}_{t}+\varepsilon_{t}$$ (2)

Where θ_i_ is a vector for long-run coefficients. It is here expected that θ_1_ > 0 and θ_1_> θ_2_ because an increase in economic growth will have a higher effect on consumption expenditure than a decline in economic growth. To account for the asymmetry impacts of income growth on consumption expenditure, we have included EG_t_^+^ and EG_t_^-^ in Eq 2 to represent the positive changes and negative changes in economic growth respectively. In this case, EG_t_^+^ and EG_t_^-^ depict the partial sum of the changes in EG_t_ such that.


$${\mathrm{EG}}_{t}{}^{+}=\mathrm{POS}{\left(\mathrm{EG}\right)}_{t}=\sum_{i=1}^{i}\Delta EG_{t}^{+}=\sum_{i=1}^{i}\max(EG_{i,0})\;\mathrm{and}$$

$${\mathrm{EG}}_{t}{}^{‐}=\mathrm{NEG}{\left(\mathrm{EG}\right)}_{t}=\sum_{i=1}^{t}\Delta EG_{t}^{-}=\sum_{i=1}^{t}\min(EG_{i,0})$$

From Eq 3, the long-run relationship between positive shocks in economic growth and consumption expenditure is depicted by θ_1_, while θ_2_ shows the long-run relationship between the negative shocks in economic growth and consumption expenditure. Besides, we anticipate that θ_1_ > θ_2._

The unrestricted error correction form of Eq 2 can be modeled as follows [32, 33, 55]:

$$\Delta CE_{t}=\alpha_{0}+\alpha_{1}CE_{t‐1}+\alpha_{2}POS(EG{)}_{t‐1}+\alpha_{3}NEG(EG{)}_{t‐1}+\alpha_{4}IQ_{t‐1}+\alpha_{5}UE_{t‐1}+\sum_{i=1}^{n}\beta_{1}\Delta {CE}_{t-i}+\sum_{i=0}^{n}\beta_{2}\Delta POS{\left(EG\right)}_{t-i}+\sum_{i=0}^{n}\beta_{3}\Delta NEG{\left(EG\right)}_{t-i}+\sum_{i=0}^{n}\beta_{4}\Delta {IQ}_{t-i}+\sum_{i=0}^{n}\beta_{5}\Delta {UE}_{t-i}+\mu_{t}$$ (3)

### 3.3 Estimation process

To estimate the NARDL model as depicted by Eq 3, we first employed the Augmented Dickey-Fuller unit root test to determine the order of cointegration of the variables. While ARDL is appropriate for variables with different orders of integration i.e., I(0) and I(1), it is limited when it comes to the I(2) series. Thus, testing for unit root is necessary to avoid estimating spurious regression. Second, to correctly estimate Eq 3, we determined the lag length with the help of the Akaike Information Criterion (AIC). Third, we employed the bound testing cointegration method [57] to test for the existence of long-run nexus among the variables for both linear and nonlinear specification of Eqs 1 and 2 respectively, and Shin’s approach [58] in unrestricted error correction model i.e., Eq 3. Fourth, we derived the cumulative dynamic multiplier of 1% positive and negative changes in economic growth, to estimate the long-run asymmetric impact of changes in economic growth on consumption expenditure. Finally, we apply the Granger causality approach [59] to examine the causal nexus among the variables.

## 4.0 Findings and discussions

### 4.1 Unit root test

To determine whether the series is stationary or not, this study employed the augmented Dickey-Fuller test, i.e., the ADF test [60], and compared the results with the Phillips and Perron test, i.e., PP test [61]. The stationarity test is an important step in regression analysis to avoid generating spurious regression results. The ADF test findings shown in Table 2 confirm that CE, IQ, and UE are stationary at level. However, EG contains unit root at level, but it is stationary at first difference. Thus, the series CE, IQ, and UE are integrated of order 0, i.e., I(0), while the series EG is integrated of order 1, i.e., I(1). To substantiate the ADF test results, we carried out the PP stationarity test which is said to be more powerful than the ADF test [32]. The PP results in Table 2, are consistent with the ADF test results. These results allow us to apply the NARDL model for no series that is integrated of order I(2).

**Table 2:** Series stationarity tests.

Variable	ADF test statistic		PP test statistic	
	Level	1^st^ Difference	Level	1^st^ Difference
CE	-4.7292***	-5.0108***	-4.3640***	-9.2201***
EG	-1.9427	-6.3082***	-1.7593	-6.2903***
IQ	-5.0613***	-5.6512***	-4.6879***	-13.3109***
UE	-3.5171*	-3.7567***	-3.8709***	-9.1035***

Note

** and *** indicate statistically significant at 5% and 1% respectively.

### 4.2 Lag length determination

Time series estimation is sensitive to lag length. Therefore, it is necessary to determine the optimal lag before running a regression. Table 3 shows the results of lag order selection criteria. Following the Akaike information criterion (AIC), when CE is the dependent variable, the optimal lag is two.

**Table 3:** VAR lag order selection criteria.

Endogenous variables: CE						
Exogenous variables: C EG IQ UE						
Lag	LogL	LR	FPE	AIC	SC	HQ
0	-74.59199	NA*	16.08367	5.613714	5.804029*	5.671895*
1	-74.24025	0.577871	16.87800	5.660018	5.897911	5.732744
2	-72.40492	2.884083	15.94621*	5.600352*	5.885824	5.687623

* Indicates lag order selected by the criterion

### 4.3 NARDL long-run form and bounds test

Table 4 shows the estimation of long-run form and bounds test results. We observe that the calculated F-statistic, which is 7.33, is greater than the upper bound limit I(1), at a 1% level i.e., 4.37. This result is evidence that there is cointegration (long-run relationship) among the variables.

**Table 4:** ARDL long-run form and bounds test.

Dependent Variable: D(CE)				
Conditional Error Correction Regression				
Variable	Coefficient	Std. Error	t-Statistic	Prob.
C	6.392531	6.584752	0.970808	0.4032
CE(-1)*	-1.932861	0.543603	-3.555648	0.0379
EG_POS(-1)	-0.019496	1.108228	-0.017592	0.9871
EG_NEG(-1)	0.259880	1.051838	0.247072	0.8208
IQ(-1)	-4.184931	6.099286	-0.686135	0.5419
UE(-1)	1.164577	0.281881	4.131453	0.0257
D(CE(-1))	0.247849	0.365234	0.678603	0.5460
D(EG_POS)	-4.209486	1.454808	-2.893499	0.0628
D(EG_POS(-1))	-8.525093	2.386922	-3.571585	0.0375
D(EG_POS(-2))	-1.340253	2.770936	-0.483683	0.6617
D(EG_POS(-3))	7.055739	1.944731	3.628131	0.0360
D(EG_NEG)	3.645085	1.813456	2.010022	0.1380
D(EG_NEG(-1))	3.923847	2.042104	1.921473	0.1504
D(EG_NEG(-2))	-4.980706	1.754679	-2.838528	0.0657
D(EG_NEG(-3))	-7.198717	3.022139	-2.381994	0.0974
D(IQ)	2.052638	4.962288	0.413648	0.7069
D(IQ(-1))	5.109531	5.640301	0.905897	0.4318
D(IQ(-2))	-14.03333	3.596815	-3.901600	0.0299
D(IQ(-3))	-7.471865	2.363949	-3.160755	0.0508
D(UE)	0.116548	0.168035	0.693597	0.5378
D(UE(-1))	-0.636641	0.171041	-3.722161	0.0338
D(UE(-2))	-0.139274	0.141980	-0.980942	0.3990
* p-value incompatible with t-Bounds distribution.				
Levels Equation				
Case 2: Restricted Constant and No Trend				
Variable	Coefficient	Std. Error	t-Statistic	Prob.
EG_POS	-0.010087	0.571950	-0.017636	0.9870
EG_NEG	0.134454	0.553401	0.242959	0.8237
IQ	-2.165148	3.291425	-0.657815	0.5576
UE	0.602515	0.245516	2.454076	0.0913
C	3.307290	2.738357	1.207764	0.3137
EC = CE—(-0.0101*EG_POS + 0.1345*EG_NEG -2.1651*IQ + 0.6025*UE + 3.3073)				
F-Bounds Test		Null Hypothesis: No levels relationship		
Test Statistic	Value	Signif.	I(0)	I(1)
			Asymptotic: n = 1000	
F-statistic	7.339637	10%	2.2	3.09
K	4	5%	2.56	3.49
		2.5%	2.88	3.87
		1%	3.29	4.37
Actual Sample Size	25		Finite Sample: n = 30	
		10%	2.525	3.56
		5%	3.058	4.223
		1%	4.28	5.84

Note: The variables EG_POS(-1), EG_NEG(-1), IQ(-1), UE(-1), D(CE(-1)), D(EG_POS), D(EG_POS(-1)), D(EG_POS(-2)), D(EG_POS(-3)), D(EG_NEG), D(EG_NEG(-1)), D(IQ), D(IQ(-1)), D(IQ(-2)), D(IQ(-3)), D(UE), D(UE(-1)), and D(UE(-2)), are system generated and they refer to the short-run changes (increase or decrease) of the primary variables defined under equation one above.

In view of Eq 2, Table 4 results show that in the short-run the coefficients of D(EG_POS(-1)), D(EG_NEG(-2)), D(IQ(-2)), and D(UE(-1)) are all statistically significant at 5% level, while the rest of the coefficients are not statistically significant. In the long-run, the coefficients of UE(-1) and CE(-1) are both statistically significant at a 5% level. Since some of the variables in the NARDL estimation are not significant, for forecasting and exploring the long-run asymmetric relationship, a parsimonious model based on NARDL findings needs to be estimated. Hence, we estimate a stepwise regression based on Table 4 results.

### 4.4 Stepwise estimation

The upper part of Table 4 shows a form of parsimonious model which has been depicted by the AIC criteria. Table 5 shows the results of Stepwise Regression when CE is the dependent variable. The parsimonious estimation results show that in the long-run changes in CE are explained by CE(-1) and IQ(-1). In the short-run, changes in CE are significantly accounted for by D(CE(-1)) and D(EG_POS(-1)). Besides, our parsimonious results fulfill the anticipation that θ_1_, θ_2_ > 0. The non-significance of EG_POS(-1) and EG_NEG(-1) in the long-run, while IQ(-1) is significant in the long-run is very revealing. This suggests that in the case of Tanzania, unlike the traditional expectation that higher levels of economic growth will deliver increased consumption expenditure and so alleviate consumption deprivation both in the long and short-run, on the contrary, in the long-run increased economic growth alone cannot significantly lower consumption deprivation poverty. Instead, in the long-run, unless income inequality is fundamentally managed, increased economic growth will not lead to poverty alleviation because increased benefits of economic growth will be eroded by growing income inequality as well as unemployment.

**Table 5:** Stepwise regression.

Dependent Variable: D(CE)				
Variable	Coefficient	Std. Error	t-Statistic	Prob.*
C	6.058014	1.926842	3.144012	0.0053
CE(-1)	-1.361729	0.263318	-5.171417	0.0001
EG_POS(-1)	0.194350	0.486186	0.399743	0.6938
EG_NEG(-1)	0.681684	0.658160	1.035742	0.3133
IQ(-1)	-3.625603	1.829499	-1.981746	0.0622
UE(-1)	-0.024399	0.096638	-0.252483	0.8034
D(CE(-1))	0.579567	0.184611	3.139390	0.0054
D(EG_POS(-1))	-2.445476	1.160555	-2.107160	0.0486
R-squared	0.664271	Mean dependent var		-0.014847
Adjusted R-squared	0.540581	S.D. dependent var		5.228283
S.E. of regression	3.543752	Akaike info criterion		5.609444
Sum squared resid	238.6054	Schwarz criterion		5.993396
Log likelihood	-67.72749	Hannan-Quinn criter.		5.723613
F-statistic	5.370468	Durbin-Watson stat		2.303003
Prob(F-statistic)	0.001621			
	Selection Summary			
Added D(CE(-1))				
Added D(EG_POS(-1))				

*Note: p-values and subsequent tests do not account for stepwise selection.

### 4.5 Wald coefficient diagnostic test

Wald test is necessary to enable us to determine long-run symmetry. The stepwise regression results help us to develop estimation commands as shown in the upper part of Table 6, which is a representation of stepwise regression results in Table 5. The estimation command identifies the long-run coeffects of the independent variables i.e., C(3) to C(8). The Wald test for the long-run asymmetry will therefore seek to identify if C(3) = C(4). Table 7 summarizes the Wald test results.

**Table 6:** Estimation command, equation, and substituted coefficients.

Estimation Command: STEPLS(METHOD = UNI, FTOL = 0.05) D(CE) C CE(-1) EG_POS(-1) EG_NEG(-1) IQ(-1) UE(-1) @ D(CE(-1)) D(EG_POS) D(EG_POS(-1)) D(EG_POS(-2)) D(EG_POS(-3)) D(EG_NEG) D(EG_NEG(-1)) D(EG_NEG(-2)) D(EG_NEG(-3)) D(IQ) D(IQ(-1)) D(IQ(-2)) D(IQ(-3)) D(UE) D(UE(-1)) D(UE(-2)) Estimation Equation: D(CE) = C(1) + C(2)*CE(-1) + C(3)*EG_POS(-1) + C(4)*EG_NEG(-1) + C(5)*IQ(-1) + C(6)*UE(-1) + C(7)*D(CE(-1)) + C(8)*D(EG_POS(-1)) Substituted Coefficients: D(CE) = 6.0580141713–1.36172911395*CE(-1) + 0.194349519074*EG_POS(-1) + 0.681684343248*EG_NEG(-1) - 3.6256028089*IQ(-1) - 0.0243992997058*UE(-1) + 0.57956680629*D(CE(-1)) - 2.44547606043*D(EG_POS(-1))

**Table 7:** Wald test.

Test Statistic	Value	Df	Probability
t-statistic	-1.853106	19	0.0795
F-statistic	3.434002	(1, 19)	0.0795
Chi-square	3.434002	1	0.0639
Null Hypothesis: C(3) = C(4)			
Null Hypothesis Summary:			
Normalized Restriction (= 0)		Value	Std. Err.
C(3)—C(4)		-0.487335	0.262983

Restrictions are linear in coefficients.

**Fig 2:**
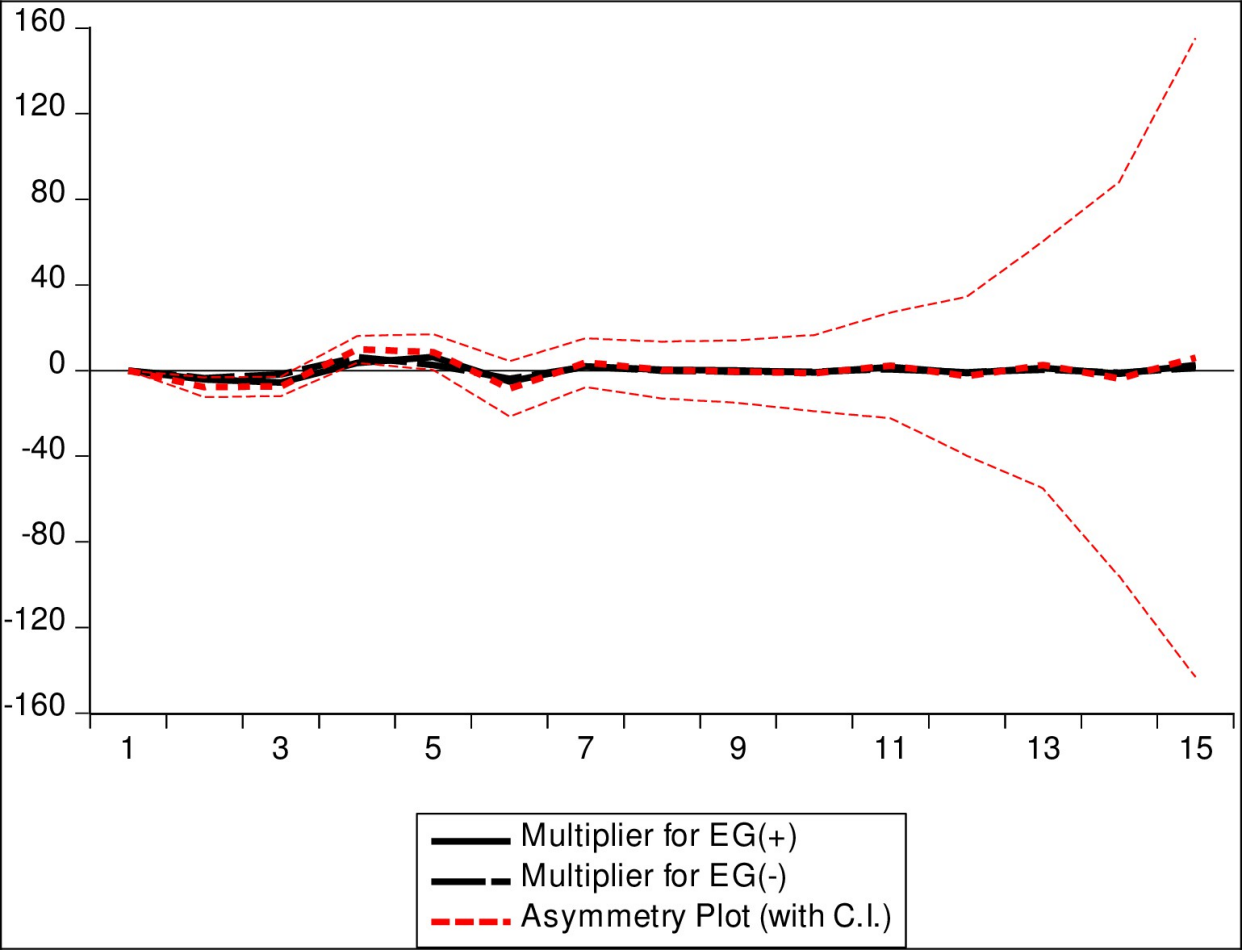
Multiplier graph for EG(Pos) and EG(Neg).

Table 7 shows Wald test coefficient diagnostic results. The decision criterion is that if we fail to reject the null hypothesis i.e. if the variables are equal, then we conclude that there is long-run asymmetry. Table 7 results affirm that the probability, at a 5% critical value level, could not reject the equality of C(3) and C(4) which means there is long-run symmetry. These results are further enriched by plotting the NARDL multiplier effects curve, i.e., *Fig 2: Multiplier graph for EG(Pos) and EG(Neg)*. The continuous black line in the middle of the chat shows how the consumption expenditure (CE) adjusts due to positive shocks in economic growth (EG). The black dotted line (overlapping the black line) shows how CE responds to negative shocks in the EG. The pattern of these two lines gives the impression that the dependent variable responds almost in the same way to the positive and negative shocks in the regressors. The bold dash-dotted red line in the middle of the chat is the asymmetry plot; it depicts the difference between the dynamic movements of positive and negative changes in the regressor. The asymmetry plot lies between the upper and lower bounds (i.e., the small dashed red line) of the 95% confidence interval. Since part of the horizontal zero line lies outside the critical bound region, then the figure affirms the existence of long-run asymmetry.

### 4.6 Causality test

Since cointegration exists among the variables, vector error correction (VEC) causality can be estimated. The causality estimation results, as summarized in Table 8, forms a basis for inferring long-run and short-run causality among the variables: In the short-run, there is bidirectional causality between consumption expenditure and economic growth; unidirectional causality from income inequality to consumption expenditure; and from unemployment to consumption expenditure. In the long-run, there is unidirectional causality from consumption expenditure to economic growth, and from consumption expenditure to unemployment. These results are consistent with the NARDL findings.

**Table 8:** Causality test—t-statistic approach.

Dependent variable	Independent Variable	Coefficient	t-Statistic	Causality
D(CE)	C(1) = CE(-1)	-1.1511***	-4.660440	Long-run causality
	C(2) = D(CE(-1))	0.6777***	2.935711	Short-run causality
	C(3) = D(EG(-1))	-1.1569*	-1.662956	Short-run causality
	C(4) = D(IQ(-1))	2.8003*	1.790270	Short-run causality
	C(5) = D(UE(-1))	-0.1678*	-1.793193	Short-run causality
D(EG)	C(7) = CE((-1)	-0.1410*	-1.739705	Long-run causality
	C(8) = D(CE(-1))	0.1350*	1.782344	Short-run causality
	C(9) = D(EG(-1))	-0.4418*	-1.935076	Short-run causality
D(IQ)	C(16) = D(IQ(-1))	-0.3926**	-2.078803	Short-run causality
D(UE)	C(19) = CE(-1)	-1.1720*	-1.792856	Long-run causality

Note

*, **, and *** indicate statistically significant at 10%, 5% and 1% respectively.

### 4.7 Diagnostic tests

To assess the robustness of our findings, three key tests were carried out: serial correlation LM test, Heteroskedasticity, and normality test. The results are summarized and presented in Table 9. Since the p-value corresponding to the Serial correlation LM test is bigger than 0.05, we fail to reject the null hypothesis and conclude that our model is not suffering from residual autocorrelation. Likewise, the F-test of heteroskedasticity test has a significance of 0.942 which is greater than 0.05. Thus, we reject the null hypothesis and affirm that the residuals are homoscedastic. Finally, the p-value for the normality test is greater than 0.05; we fail to reject the null hypothesis. Therefore, the residuals are multivariate normal.

**Table 9:** Diagnostic tests.

	Null Hypothesis (Ho)	F-statistic	p-value	Remarks
Serial correlation LM test	There is no problem with serial correlation	2.3237	0.4208	Fail to reject Ho
Heteroskedasticity	The residuals are homoscedastic	0.3426	0.9420	Fail to reject Ho
Normality test	Residuals are multivariate normal	Jarque-Bera: 1.4486	p-value: 0.4846	Fail to reject Ho

### 4.8 Model stability test

**Fig 3:**
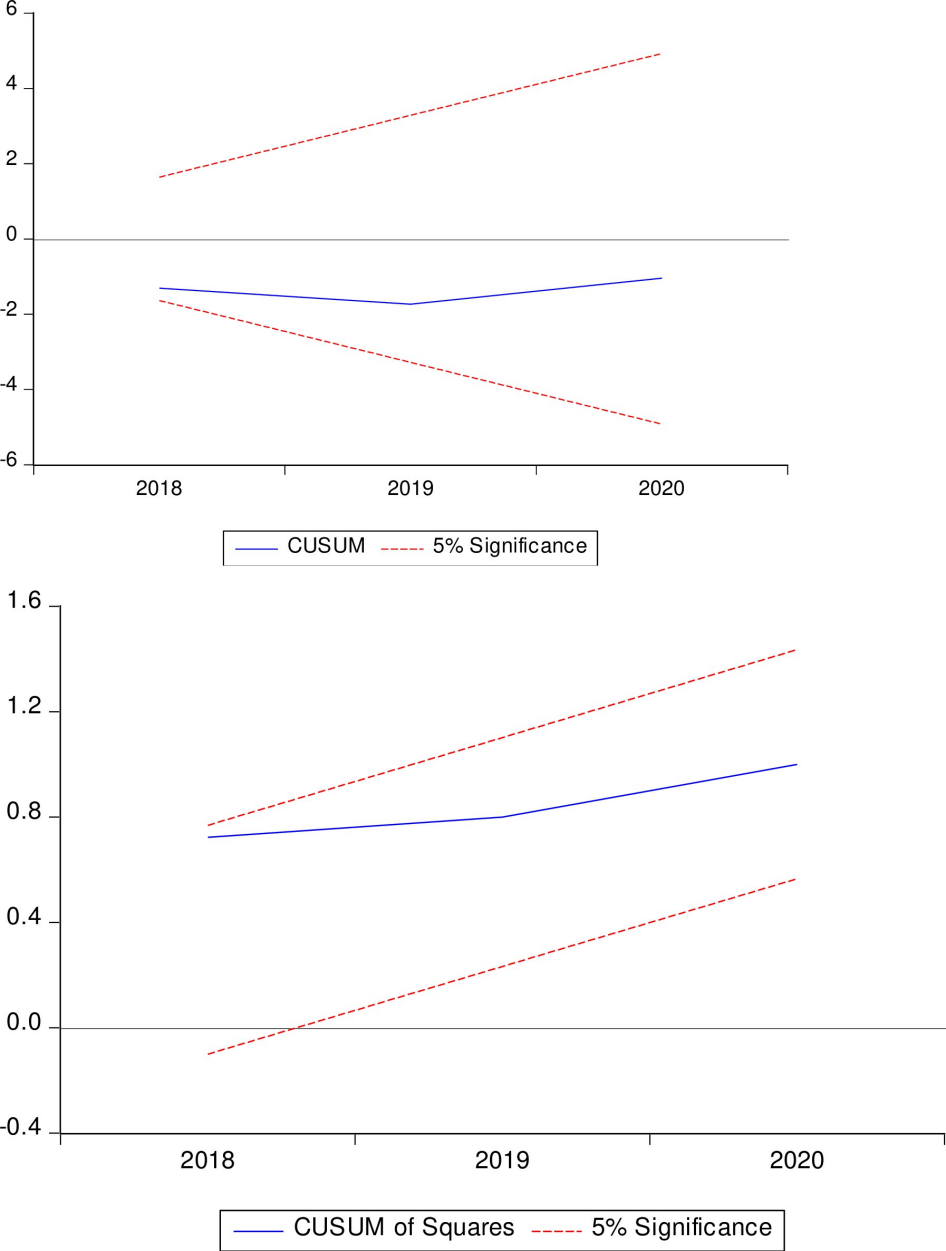
Model stability: CUSUM & CUSUM square test.

To test for model stability, we employed CUSUM and CUSUM Square Test and the results are depicted by *Fig 3: Model stability: CUSUM & CUSUM Square Test*. The upper part of the figure represents the CUSUM line chart, and the lower part represents the CUSUM Square line chart. The two charts are based on accumulated residuals and aggregate residual squares, respectively. While the CUSUM detects systematic modifications in regression coefficients, the CUSUM Square test detects drastic changes in the permanence of the regression coefficients. The dotted red lines in both charts represent the upper and lower boundary (i.e., control line) of the CUSUM and CUSUM Square chats. The blue lines in the upper chart and lower chart represent CUSUM and CUSUM Square respectively. Therefore, the figure suggests that the model is stable because both the CUSUM and CUSUM Square line charts lie within the 5% specified critical boundary.

## 5.0 Concluding remarks and policy implications

This paper analyzed the impacts of economic growth on per capita consumption expenditure in Tanzania. To capture the long-run and short-run asymmetric relationship between consumption expenditure and economic growth, we adopted the nonlinear autoregressive distributed lag (NARDL) model and Wald test. Further, to explore the causal relationship among the variables, (i.e., per capita consumption expenditure, economic growth, income inequality, and unemployment), we employed the Granger causality test approach. The estimated results confirmed the presence of long-run and short-run asymmetric behavior of economic growth. In the long-run, changes in income inequality are significant and they account for changes in consumption expenditure. In the short-run, an increase in economic growth is associated with increasing consumption expenditure and vice versa. Besides, the causality test confirms a bidirectional causality between consumption expenditure and economic growth in the short-run. Likewise, in the short-run, there is unidirectional causality from income inequality to consumption expenditure, and from unemployment to consumption expenditure. In the long-run, the study generated evidence of unidirectional causality from consumption expenditure to economic growth, and from consumption expenditure to unemployment. The causality results are consistent with the NARDL results.

From a policy viewpoint, this research demonstrates some key conclusions, pointing to the applications of the findings: First, the evidence shows that only income inequality is significant in the long-run, and economic growth in the short-run indicates the systemic effect of inequality in the redistribution of income. It affirms that in the case of Tanzania, increased economic growth is necessary for containing consumption deprivation but in the long-run, the rising income inequality interacts with economic growth and dampens the positive poverty-alleviating impact of economic growth. Thus, policy attention should be directed to containing income inequality if increased benefits of economic growth should count significantly in reducing consumption poverty and improving populations’ wellbeing. Robust economic growth-related policies to spearhead poverty alleviation must be accompanied by strategies to alleviate income inequality. For the real poor to realize the full benefits of economic growth, it is necessary to institute policy instruments to encourage economic progress and at the same time address the appalling structures which give rise to income inequality. Strengthening collective bargaining rights among the low- and middle-income earners, promoting the adoption of living-wage policies, the introduction of stronger minimum wage law, subsidizing the provision of public goods e.g., health care and education, facilitating greater access to higher-income jobs, and promoting workers’ rights to resources ownership are some of the recommended programs to contain income inequality.

Second, the short-run bidirectional causality between consumption expenditure and economic growth indicates that policies to promote economic growth will lead to increased consumption expenditure and vice versa. The short-run unidirectional causality from income inequality and unemployment to consumption expenditure underscores the need for policy instruments to contain both income inequality and unemployment, promote increased consumption expenditure, and improve the population’s wellbeing. High- and persistent-income inequality and unemployment erode individuals’ ability to access necessities of life due to lack of necessary income and so deepens deprivation poverty. For instance, unemployment is associated with limited autonomous consumption, which is a mere subsistence, and it does not provide multiplying effects for improved wellbeing.

Third, the evidence that in the long-run income inequality influence the level of consumption expenditure and in turn consumption expenditure Granger causes the level of economic growth and unemployment implies that policies to contain income inequality in Tanzania will, in the long-run, carb unemployment and promote economic growth and consumption expenditure. In the long-run, income inequality has a significant sapping effect on economic growth. Thus, since income inequality in Tanzania is mostly manifested in the agriculture sector, (as compared to other sectors), and since the sector hosts the majority of the country’s poor, for poverty reduction initiatives to be effective, concerted efforts must be focused on transforming agriculture sector to promote income and employment within the sector. Improved farmers’ access to credit facilities, regular hands-on training on improved farming and animal husbandry, processing of agricultural products in situ for value addition, access to simple technologies to reduce post-harvest losses, and improved access to market and resources ownership are some of the recommended strategies to transform the agriculture sector. Besides, policy instruments promoting investment in the agriculture sector are significant for poverty reduction and improved wellbeing. In the case of Tanzania, promoting economic growth, without a simultaneous implementation of robust policies to tackle income inequality which perpetuates poverty at the grassroots, will not deliver the desired long-run results.

The main limitation of our study is the availability of data, i.e., there are no reliable sources of data, for the variables in question, for the period before 1991. As a result, the research had to use a relatively small sample, i.e., data for the period 1991–2020. To address this problem the authors settled for the NARDL methodology because it is a more reliable method in the presence of small samples.

Finally, for future research examining the growth-poverty dilemma in developing countries, we recommend research in the following areas. First, exploration of other factors which are deemed to contribute to the growth-poverty nexus. These include demographic factors (e.g., the influence of rapidly increasing population and its quality on consumption poverty), methodological challenges (e.g., the limited budget and lack of adequate skills needed for data collection, processing, and management by countries’ bureau of statistics), and stagnation in the agricultural sector. For instance, in Tanzania even though the sector hosts over 74% of the population, it is facing multiple challenges such as limited public expenditure, poor access to private land ownership, lack of access to credit facilities, etc. Second, exclusive investigation of the growth-poverty dilemma from an ethical perspective is under-researched in developing nations. Such an investigation will provide corroborating evidence on the role of ethics in the ongoing efforts to account for the evolution of poverty, thereby addressing the prevailing scenario of growth without prosperity in developing nations. For instance, scholars may consider researching areas such as evaluating commitments of multinational corporations on poverty alleviation, stakeholders’ perceptions about the effectiveness of corporate social responsibility as poverty alleviation and wellbeing improvement strategy, and ethical innovation to poverty reduction. Third, we recommend a sectoral analysis to depict the effects of economic growth by sectors and the sectoral impact on poverty alleviation.
